# MedTech start-ups: A comprehensive scoping review of current research trends and future directions

**DOI:** 10.1371/journal.pone.0307959

**Published:** 2024-08-06

**Authors:** Olga Kalinowska-Beszczyńska, Katarzyna Prędkiewicz

**Affiliations:** 1 Division of Public Health & Social Medicine, Medical University of Gdansk, Gdansk, Poland; 2 Finance Department, Wroclaw University of Economics and Business, Wroclaw, Poland; West Pomeranian University of Technology, POLAND

## Abstract

Medical start-ups (MedTech) significantly contribute to the development and commercialization of innovative healthcare solutions, driving advancements in technology, enhancing treatment effectiveness, and supporting public health. This study explores the main themes and concepts related to MedTech start-ups, examines the research methods used, and identifies major gaps in the literature. A scoping literature review was performed by searching the Scopus, PubMed, and Web of Science databases for publications from 2012 to 2023, focusing on MedTech start-ups in titles, abstracts, and keywords. References were analyzed using the Bibliometrix package in R, and a coupling network analysis was conducted, visualizing results on a Coupling Map to identify key research themes and gaps. The research identified 480 unique articles on MedTech start-ups. After removing duplicates and following a PRISMA-based assessment, 79 articles were included in the review. The studies predominantly focused on organizations, including start-ups and Venture Capital funds (46%). Most articles (60%) used qualitative methods, 25% employed mixed methods, and 15% used quantitative methods. Geographically, 63% of articles focused on a single country, primarily the USA (35%), followed by Iran, Sweden, Switzerland, China, and Japan (2–4% each). Coupling analysis identified five topic clusters: crowdfunding for medical research, innovation in medical technology, new product development, digital start-ups, and the venture capital industry. This review highlights the significant role of MedTech start-ups in advancing healthcare innovations despite challenges like regulatory hurdles and high capital requirements. The literature emphasizes the importance of collaboration among universities, industry, and government for successful commercialization. The geographic concentration in the USA indicates a need for more inclusive research. Crowdfunding and venture capital emerge as crucial funding sources, suggesting strategies to mitigate risks and enhance innovation success.

## Introduction

Medical start-ups (MedTech) are at the forefront of addressing the numerous challenges facing modern healthcare systems. As the global population grows and ages, healthcare systems worldwide are under increasing pressure to provide high-quality care with limited financial resources. Medical start-ups offer promising solutions by enhancing the efficiency of medical services, spanning prevention, diagnosis, treatment, and patient management.

MedTech start-ups can be defined as companies that develop and commercialize new medical technologies and innovations. These innovations can be categorized into two primary areas: In Vitro Diagnostics (IVD) and Medical Devices. IVD includes tools such as self-tests for pregnancy or genetic tests for cancer, which provide critical health information from biological samples [1]. Medical Devices cover a diverse array of applications, from cardiology equipment to orthopedic implants, each designed to address specific medical needs [2].

From an economic perspective, the MedTech industry is a significant driver of growth and innovation. It has consistently outperformed the Standard&Poor index (S&P index) by 15% over the past three decades [3]. The S&P 500 index constitutes a financial metric that quantifies the equity performance of 500 prominent corporations traded on American stock exchanges. It is commonly esteemed as a primary indicator of the aggregate market dynamics in the United States. The MedTech industry’s sustained outperformance of this index highlights its robust growth and resilience. Additionally, the industry is projected to reach a market volume of USD 719.20 billion by 2028, with a compound annual growth rate (CAGR) of 4.73% between 2023 and 2028 [4]. This growth is fuelled by advancements in technologies such as Artificial Intelligence (AI), big data analytics, and wearable devices, alongside demographic shifts like aging populations and increasing prevalence of chronic diseases, rising demand for personalized medicine [5], and finally patient education and engagement [6].

Venture Capital (VC) investment in MedTech start-ups has also surged, with a compound annual growth rate of 29% between 2017 and 2021. Investors are particularly drawn to innovations in AI and robotics, which hold significant promise for transforming healthcare delivery [7]. Despite this interest, MedTech start-ups face numerous challenges, including stringent regulatory environments, complex market dynamics, and high capital requirements for product development and commercialization [3].

In this context, the aim of this paper is to identify and evaluate the current state of academic literature on MedTech enterprises by examining the types of conducted research, key themes discussed, and significant findings, as well as suggesting potential avenues for future research. Specifically, we seek to address the following research questions:

1. What are the main themes and concepts related to MedTech start-ups?
2. What research methods are employed in studies concerning MedTech enterprises?
3. What are the major gaps in the literature regarding MedTech start-ups?
4. What are the emerging trends and future directions in the research and development of MedTech start-ups?

Through a scoping review of existing studies, we aim to provide a comprehensive overview of the MedTech start-up landscape and offer insights that can inform future research, policy-making, and practical applications within the industry.

## Materials and method

The scoping approach was selected as the most appropriate form of literature review. It allows mapping of the body of literature on a topic area [8] by systematically searching for, selecting, and analysing relevant scholarly articles to identify the key concepts, main sources, and research gaps in the literature.

The applied protocol is aligned with the guidelines provide by Peters et at., [9, 10] and includes the following sections: title; background; objectives; inclusion criteria; concepts; context; searching; extracting and charting the results; discussion; conclusion and implication for research and practice. In essence, this protocol outlines the plan for conducting a scoping review, which involves examining scholarly literature to comprehend a topic, pinpoint gaps in knowledge, and identify potential avenues for future research.

The goal of our scoping review was twofold: firstly, to comprehensively assess the scope, diversity, and characteristics of research endeavours; and secondly, to identify areas where the current literature lacks investigation. There were two core research themes that our work specifically focuses on. The first theme centred around medical innovation, considering various aspects related to commercialization of new products such as: acceptance, adaptation, and evaluation of technology in the healthcare sector. The second theme revolves around MedTech start-ups, where we explored factors that impact their creation, growth and potential setbacks, including financing and fundraising opportunities.

To identify relevant papers, we formulated a set of search terms and keywords, including: "medtech," "start-up," "medical innovation," "commercialization," "success factor," and "financing" to be searched in the titles, keywords, and abstracts of potentially relevant articles. Further, we have exhaustively utilized those terms and words in different configurations. The comprehensive list is presented below:

(medtech AND start-up) OR (medical AND innovation AND start-up) OR (medical AND innovation AND start-up AND commercialization) OR (commercialization AND of AND medical AND technology AND start-up) OR (medical AND start-up AND success AND factors) OR (medtech AND start-up AND financial AND constraints) OR (medtech AND start-up AND fundrising) OR (medical AND technology AND commercialization AND smes) OR (medical AND start-up AND failure) OR (medtech AND innovation AND financing) OR (ehealth AND start-up) OR (medical AND venture AND capital) OR (medical AND crowdfunding)

The initial screening of articles was based on the following criteria:

- Must be a research study.
- No restrictions on the geographic region.
- Written in English.
- Published between 2012 and 2022.
- Appeared in a peer-reviewed journal.
- Indexed in Scopus, PubMed, or Web of Science (WoS).

Following the primary search, the database was refined to include articles that met the following specific criteria, which also served as search parameters:

- Focus on medical start-ups.
- Discussion on the commercialization of medical innovation.
- Exploration of financing for medical start-ups.
- Identification of success factors relevant to medical start-ups.
- Exclusion criteria applied during the selection process were:
- Contextual inconsistencies.
- Unaccepted document formats, such as notes or editorials.
- Non-English language.

In order to systematize the collected information, a spreadsheet was created. It allowed categorisation of publications according to the year of publication, study type, country of publication, subject of research, research technique, significant findings, and potential avenues for further research. At this stage of analysis we utilized the NVivo software to code the research questions, main findings and future research. The results of analysis are presented in next section.

To conduct bibliometric analysis, we applied the Bibliometrix, which is a free and open-source R tool for quantitative research in scientometrics, bibliometrics [11]. Bibliometrix proved to be an efficient tool to import, analyse and visualize bibliographic data from different sources (i.e. Scopus, Web of Science, PubMed) and can perform various types of bibliometric analyses. The web-based interface–Biblioshiny–provided interactive graphs, enabled generation of descriptive statistics, allowed coupling analysis, and performed clustering analysis. Additionally, we utilized the tool’s visualizations, such as word clouds and trend topics to present the bibliometric data.

Cluster analysis was used to identify the main thematic groups. Each of the clusters was characterised and an analysis of the main research questions, key findings, and suggested potential areas of investigation mentioned in the articles was conducted.

A coupling network analysis was conducted, and the results of community detection were plotted on a two-dimensional map known as the Coupling Map. This analysis can be conducted on three different units: documents, authors, or sources, and the strength of coupling can be measured using either the classical approach, which involves coupling by references, or a novel approach that incorporates unit contents such as keywords or terms from titles and abstracts. In our case the coupling measures were titles and impact measure global citation score, whereas clusters were labelled by title terms using unigrams (single words). On the Coupling Map, the x-axis represents the cluster centrality, measured by Callon’s Centrality index, while the y-axis measures the cluster impact through the Mean Normalized Local Citation Score (MNLCS). The Normalized Local Citation Score (NLCS) of a document is calculated by comparing the actual count of local citing items to the expected citation rate for documents published in the same year [11]. Callon’s Centrality index takes into account various factors such as the number of connections, the strength of those connections, and the positions of actors within the network. By analysing these factors, the index provides a quantitative measure of the centrality of a cluster in relation to other clusters in the network [12].

## Results and discussion

### Literature search

A total of 480 articles were identified during the search, with 126 articles in PubMed, 265 articles in WoS, and 396 articles in Scopus (the last articles search was made in May 2023). After merging the results and removing duplicate records, we obtained 480 unique articles (Table 1). The pair-comparison of databases reviled that Scopus and WoS had the highest percentage of duplicated documents (49%), while the lowest percentage was found between Scopus and PubMed (19%). Overall, we observed that PubMed had the lowest duplication rate compared to other databases included in the research. In terms of article population Scopus contained the highest number of unique articles (193), followed by PubMed (39) and WoS (36).

**Table 1:** Duplicated publications in WoS, Scopus, PubMed.

Databases	Total number of publication	Duplicated publications	% duplicated
WoS + Scopus + PubMed	480	304	63%
WoS + Scopus	444	217	49%
WoS + PubMed	316	72	23%
Scopus + PubMed	459	89	19%

Source: Authors’ elaboration

**Fig 1:**
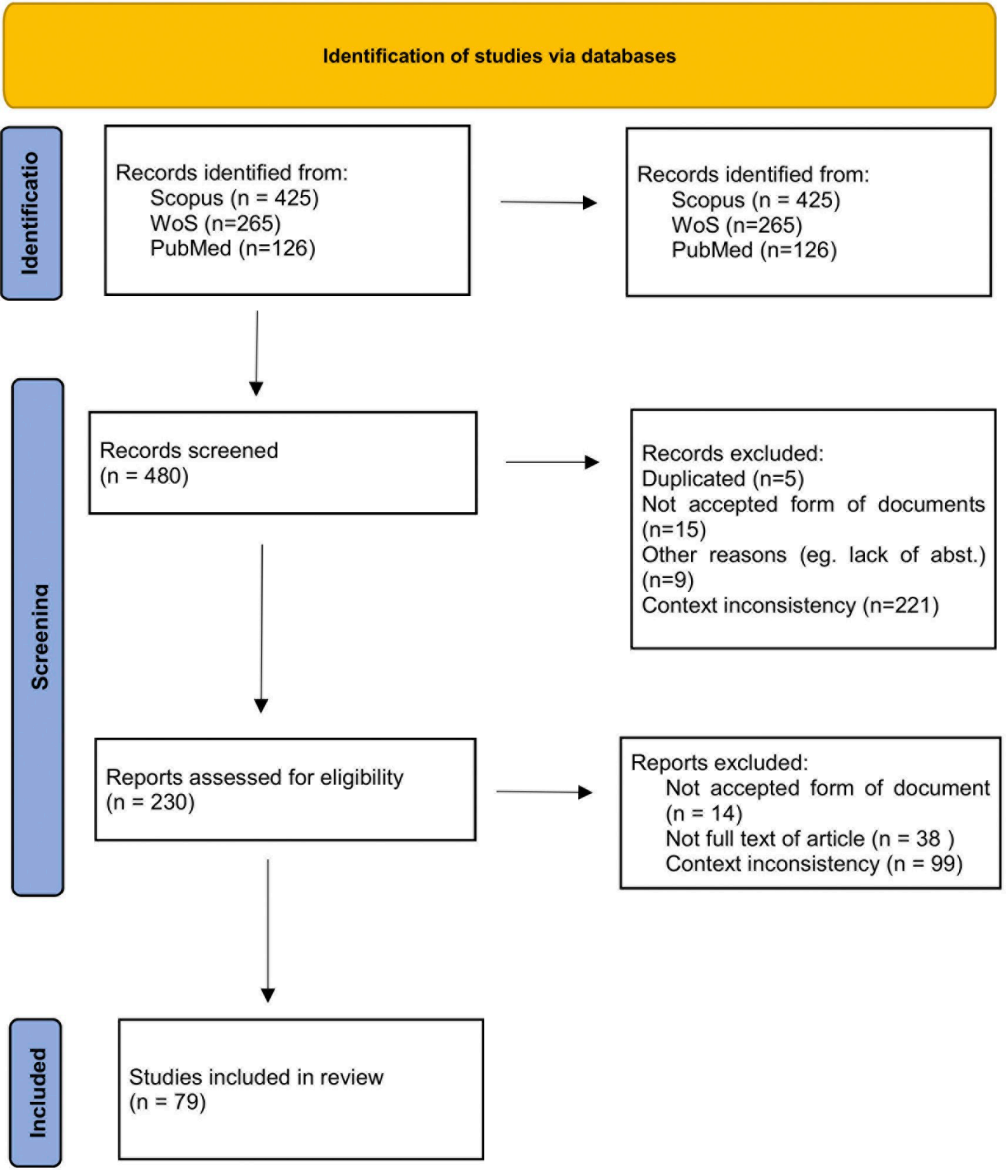
Stages followed for the scoping review/ PRISMA. Source: Authors’ elaboration based on PRISMA methodology.

To identify pertinent studies, we thoroughly assessed the abstracts of all articles using the selection criteria outlined in the PRISMA chart (Fig 1).

During the initial screening phase, we excluded 250 articles. The majority of which were deemed irrelevant due to content inconsistency such as focus on medical crowdfunding or patient-oriented donations. Other reasons for exclusion included clinical research and public health and hospital efficiency.

In the subsequent screening phase, an additional 151 articles were excluded primarily due to content inconsistency as well as other reasons such as inadequate form of the document. Ultimately, a total of 79 articles met the inclusion criteria and were included in the scoping review.

### Study characteristics

Results presented in Table 2 refer to a set of bibliometric analyses, including information about the timespan of publication, sources, document characteristics, authorship, and collaboration patterns. Thus setting a context for further investigation of the field.

**Table 2:** Bibliometrix analysis.

Description	Results
Timespan	2012:2022
Sources (Journals, Books, etc)	70
Documents	79
Average years from publication	4.17
Average citations per documents	11.43
Average citations per year per doc	2.012
References	3389
*Document contents*	
	553
Author’s Keywords (DE)	296
*Authors*	
Authors	283
Author Appearances	300
Authors of single-authored documents	11
Authors of multi-authored documents	272
*Authors collaboration*	
Single-authored documents	12
Documents per Author	0.286
Authors per Document	3.49
Co-Authors per Documents	3.7
Collaboration Index	3.94

Source: Authors’ elaboration using Bibliometrix

The analysis covered a timespan from the year 2012 to the year 2022, utilizing data from 70 different sources. The number of academic articles published in the area of med-tech start-up is relatively small compared to the attention that is given to the subject in the industry led discussions and publications.

Turning to detailed description we observe that documents under investigation were published approximately 4.17 years ago. Each document received an average of 11.43 citations, which indicated the level of scholarly attention they garnered. Furthermore, the average number of citations per year per document was found to be 2.012, indicating the ongoing impact and influence of the documents over time. The documents collectively cited a total of 3,389 references, reflecting the breadth of scholarly sources consulted.

Considering the authors engagement we identified a total of 283 authors contributed to the analysed papers, with their appearances amounting to 300 individual contributions. The majority of the documents had multiple authors, indicating a collaborative approach to research.

Further insights were gained by examining the collaboration patterns among authors. Within our sample only 12 documents were single-authored. On average, there were 3.46 authors per document. The collaboration index indicating the overall level of authors’ collaboration was calculated at the level of 3.94 points.

The applied methodology allowed us to analyse keywords used to describe the articles. Specifically, there were 553 unique additional keywords known as "KeyWords Plus (ID)," and 296 unique author-assigned keywords known as "Author’s Keywords (DE)." KeyWords Plus are words or phrases that frequently appear in the titles of an article’s references, but do not appear in the title of the article itself. They are generated automatically by special algorithms in bibliometrics’ databases. These keywords reflect the various topics and themes covered within the analysed documents.

**Fig 2:**
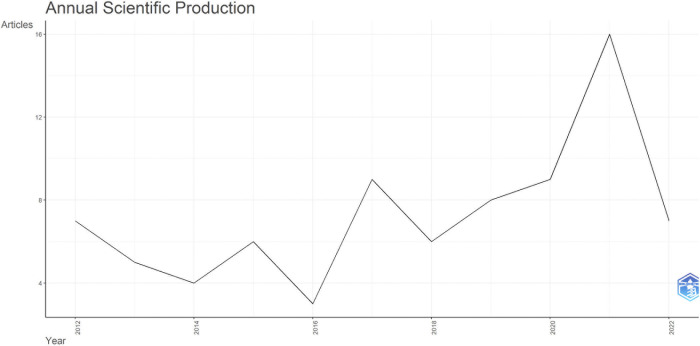
Annual scientific production for combined data. Source: Authors’ elaboration using Bibliometrix.

One may observe a growing interest of the research community in the subject of MedTech start-ups. This is illustrated by the general upward trend in the number of published research (Fig 2). The majority of articles (69%) were published between 2017 and 2022, indicating a concentrated period of research activity in this field. The average annual growth rate for the analysed period between 2012 and 2022, was observed at a steady increase of 19%.

The most active authors were Muhos (co-author of 3 articles) and Saarela (2 articles together with Muhos). Three articles focused on growth management of e-health service start-ups [13], management priorities of digital health service start-ups in California [14] and digital healthcare service start-ups case studies from Sweden [15].

The most cited document was titled “*Who takes you to the dance*? *How partners institutional logics influence innovation in young firms*” by Pahnke, Katila, and Eisenhardt [16]. Thus it might be characterised as the most widely acknowledged and highest impact paper in the sample. This study is relevant to MedTech start-ups as it reveals how the institutional logics of different funding partners (venture capitalists, corporate venture capitalists, government agencies) impact innovation, helping these start-ups strategically choose partners to enhance their innovation outcomes.

Considering the geographical location the analysed articles predominantly focused on the medical start-up topic within a single country (63%). Approximately 10% of the articles explored the subject matter in multiple countries settings, ranging from two to twelve countries. Only one article addressed the topic in the a global scale perspective. Among the single-country studies, American medical start-ups garnered the highest level of interest from researchers, accounting for 35% of all articles. Iran, Sweden, Switzerland, China, and Japan also received attention, with each country representing 2–4% of the articles. The remaining research primarily centred on various European countries, such as Belgium, France, Denmark, the Netherlands, and Spain.

There is a notable gap in the literature due to the predominance of research focused on the USA. To gain a more comprehensive understanding of the phenomenon, it is essential to conduct more diverse and inclusive studies that encompass a wider range of countries and regions.

From the research object we identified the following streams. The vast majority of papers focused on organizations, including start-ups and Venture Capital funds, accounting for 46% (n = 37) of the articles. A smaller fraction of the papers explored policies or programs related to medical start-ups, representing 9% (n = 7) of the total. Also crowdfunding campaigns gained interest as a potential source of financing for new ventures in the MedTech field received attention. Other areas of research encompassed collaboration models, industry analysis, patents, projects, and stakeholders, with each topic representing 1% of the articles. However, a significant portion, 33% (n = 27), could not be classified into any specific subject category due to their broader focus on different aspects of the med-tech industry without a clear thematic focus. We may conclude that the research predominantly focused on organizations, with start-ups and Venture Capital funds being of particular interest. While some studies explored policies, crowdfunding, and various specific aspects of the med-tech industry, a considerable number of articles covered a broader range of topics without a distinct subject classification.

The applied type of research method was also under investigation. Each article was categorized into one of three categories: qualitative, quantitative, or mixed methods. Majority of the analysed articles, accounting for 60% (n = 49), utilized qualitative research methods. Followed closely by 25% (n = 20) of the articles employed a mixed methods approach. Only 15% (n = 12) of the articles relied solely on quantitative research methods. This distribution indicates a notable preference for qualitative and mixed methods in the research conducted on the topic. Upon closer examination of the research techniques employed, we found that the case study method was the most prevalent, accounting for 26% of the articles. Additionally, among the qualitative research tools utilized, we observed in-depth interviews, natural experiments, and questionnaires to be the most popular. Only a small fraction, accounting for 9% of the articles, referred to econometric models, while 15% employed basic statistical analysis.

While investigating the sample size, we could observe that case studies employed a single entity in more than one-third of papers, and between 2 to 7 entities in another one-third of papers. Econometric models tended to rely on larger sample sizes. For example, Clarke and Kitney [17] conducted an analysis of factors determining business success based on 146 medical device start-ups. Similarly Aleksina et al., [18] examined determinants of successful crowdfunding campaigns based on 109 projects in medical research. These examples demonstrate the range of research techniques employed in the analysed articles, with case study methods being the most prevalent and other qualitative methods and a smaller proportion utilizing econometric models and basic statistical analysis. The data sources varied, the dominant forms were interviews and databases, each accounting for 21% of the articles. Authors own experiences (6%) and observations (2%) were also utilized, yet to lesser extent.

Based on these findings, we can conclude that the conducted research thus far has been predominantly reliant on limited data sources. This is often due to the challenging nature of studying start-ups, particularly within a specific sector such as MedTech. Consequently, the research samples tend to be small, making it impractical to utilize more advanced quantitative methods. The inherent complexities associated with studying start-ups, coupled with the specific focus on the med-tech industry, further contribute to the shrinking of the research sample size.

**Fig 3:**
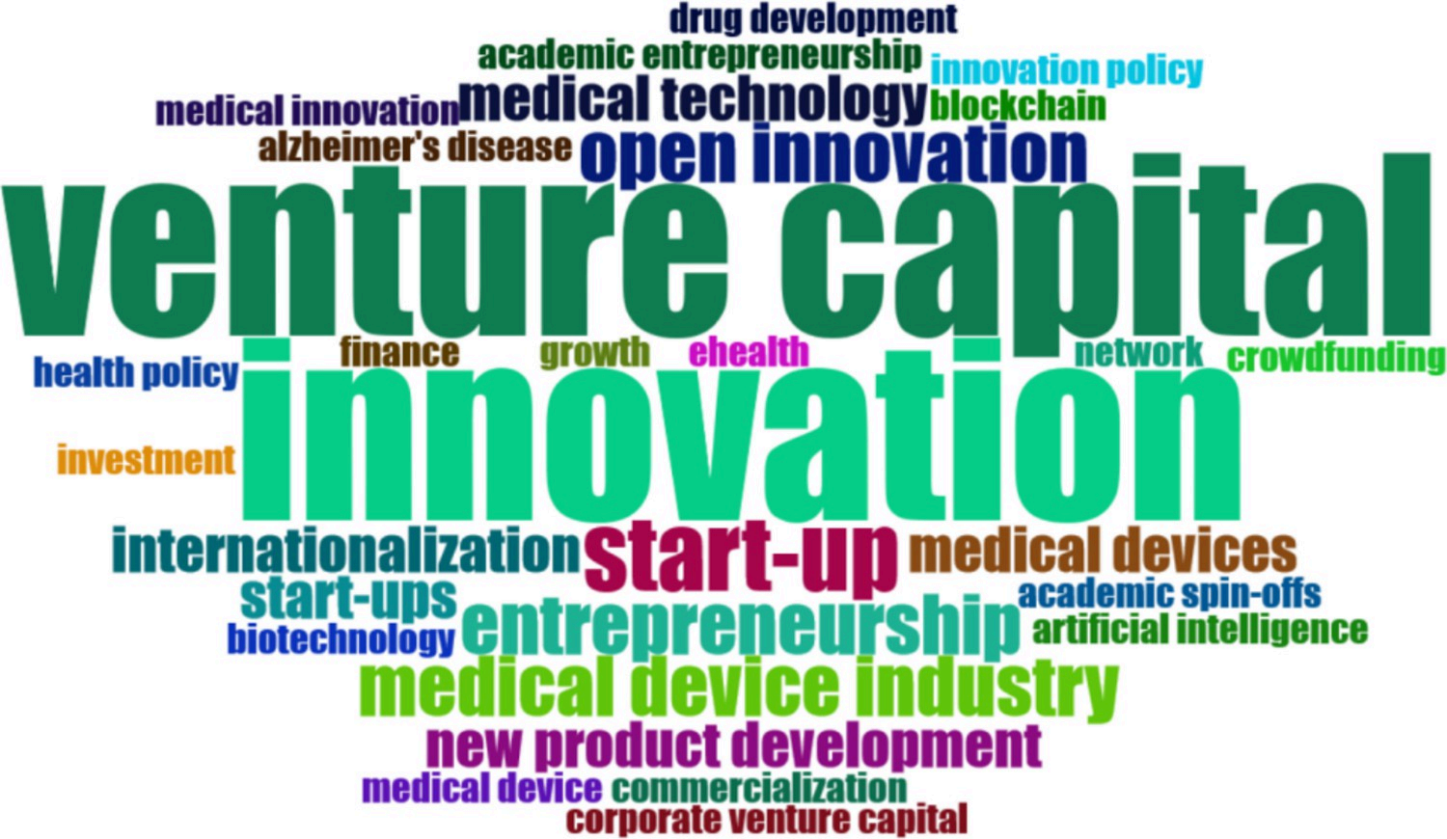
Word cloud based on authors key words. Source: Authors’ elaboration using Bibliometrix.

**Fig 4:**
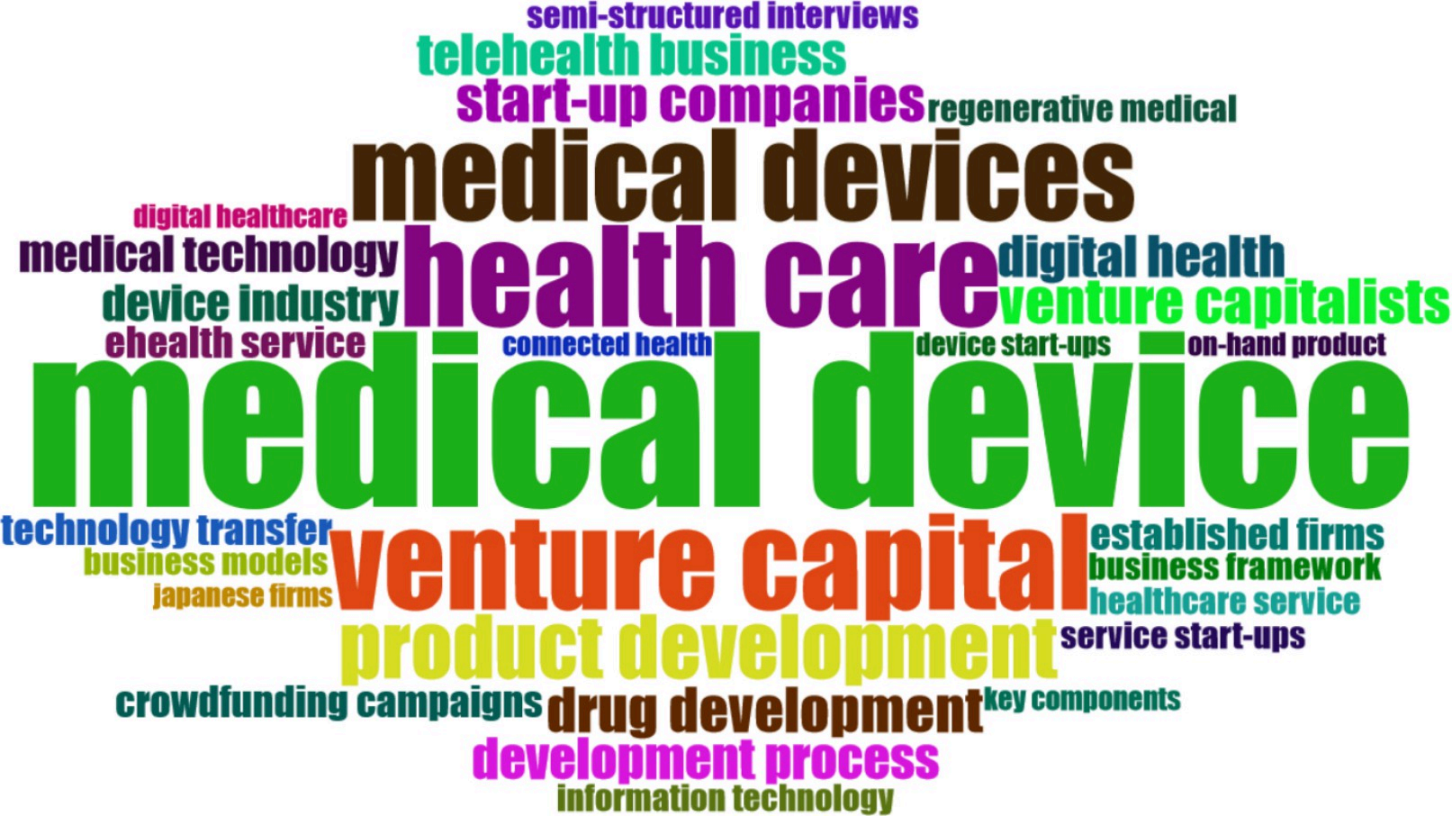
Word cloud based on abstracts. Source: Authors’ elaboration using Bibliometrix.

In order to conduct advanced bibliometric analysis Bibliometrix software was utilized, which enabled to identify such features as: word cloud analysis, thematic grouping or key research trends. The below presented figures (Figs 3 and 4) refer to tag cloud analysis conducted respectively for the keywords specified by the authors (Fig 3) and based on abstract analysis (Fig 4). Upon examining the main 30 terms derived from the authors’ keywords, we observed that the term ’Venture capital’ appeared most frequently, followed by ’innovation’ and ’start-up’ (as shown in Fig 3). Furthermore, we noted the inclusion of terms associated with the medical device industry and medical technology throughout the list.

However, the analysis of abstracts indicates different results. The two most common two-word phrases based on abstract analysis were ’medical device,’ ranking first with a frequency of 38, and the plural form ’medical devices,’ ranking third with a frequency of 20. Following closely were terms such as ’health care’ and ’venture capital,’ both with a frequency of 21. ’Start-ups’ occupied the sixth position with a frequency of 10.

By examining both perspectives, we may gain a more comprehensive understanding of the research landscape, uncovering the authors’ intended focus of research that is reflected in provided key words as well as the central concepts that emerge from the actual content of the articles mirrored in the abstracts.

The analysis demonstrates that the field of MedTech industry can be examined from two dominant viewpoints: the venture capital perspective and start-ups perspective. In both perspectives the term *‘Innovation*’ is closely related. When examining terms related to specific innovation in healthcare, the focus is given on medical devices, e-health, and drug development. Yet, the most commonly encountered term is medical device. Among commonly used terms the concept of crowdfunding was also present. However, the majority of articles related to "medical crowdfunding" were excluded during the initial and subsequent screening phases due to inconsistencies in their context. These articles predominantly focused on crowdfunding campaigns with a patient-oriented approach, whereas the presented review focuses on articles referring to crowdfunding campaigns that serve as a means of financing new medical solutions.

**Fig 5:**
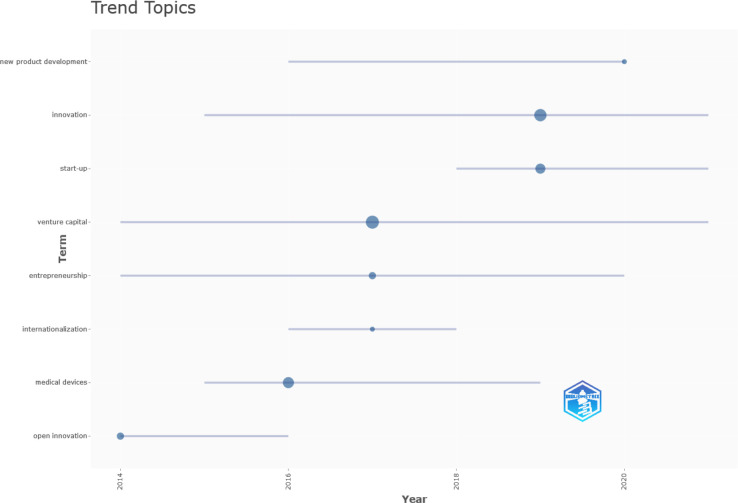
Trend topics. Source: Authors’ elaboration using Bibliometrix.

Further, utilising Bibliometrix software we were able to gain insight into the development of "trend topics" over the years of analysis using authors’ keywords (Fig 5). It is noteworthy that certain topics, such as *open innovation* and *internationalization*, have not yet captured the interest of researchers. Conversely, topics like *innovation* and *start-ups* have experienced a growing attraction, indicating their increasing significance. Additionally, one topic that has consistently remained present in research is *venture capital*.

**Fig 6:**
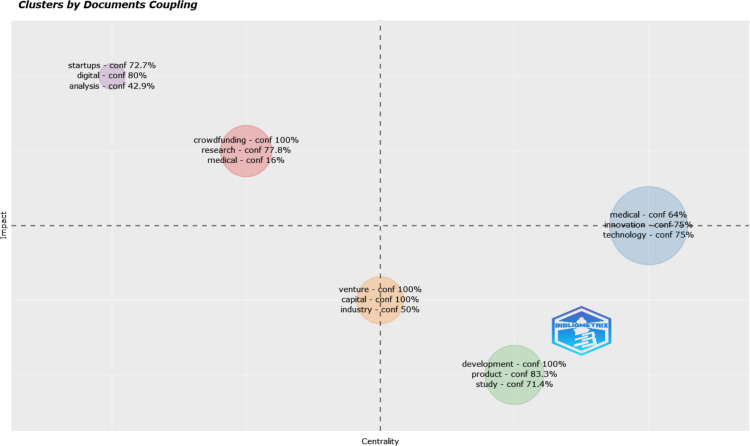
Topic clusters. Source: Authors’ elaboration using Bibliometrix.

Utilizing Bibliometrix, we have analysed the coupling between titles and identified five distinct topic clusters. We used the global citation scores to measure the impact of each cluster. It is important to note that this analysis specifically focused on unigrams, which are single-word units of analysis. Table 3 and Fig 6 summaries the cluster analysis. We may observe that Cluster 2 has the highest centrality score, followed by Cluster 3. This suggests that Cluster 2 and Cluster 3 are relatively more central and influential within the network compared to the other clusters. Considering the impact (last column), Cluster 4 has the highest impact score of all, indicating that it has a relatively higher level of impact based on global citations. Cluster 1 follows, having a moderate level of impact.

**Table 3:** Topic clusters.

Cluster	Group	freq	centrality	impact
crowdfunding—conf 100% research—conf 77.8% medical—conf 16%	1	15	1,139003182	3,244287257
medical—conf 64% innovation—conf 75% technology—conf 75%	2	23	1,472122676	2,414627135
development—conf 100% product—conf 83.3% study—conf 71.4%	3	17	1,306566533	1,922914726
startups—conf 72.7% digital—conf 80% analysis—conf 42.9%	4	10	0,988977321	5,433962264
venture—conf 100% capital—conf 100% industry—conf 50%	5	14	1,21105189	2,3735748

A concise summary of each cluster is provided below. In the following sections, a comprehensive analysis of the articles within each cluster is presented, highlighting the key findings and research themes, where *n* represents the number of articles in a cluster followed by the percentage share of articles belonging to a cluster in all the analyzed articles.

### Cluster1: Crowdfunding for medical research (n = 15; 18.99%)

The first cluster focuses on the role and impact of crowdfunding as a source of funding for health-related research projects. The articles examine the trends, importance, benefits, risks, and determinants of crowdfunding for biomedical R&D, as well as the alternative financial structures and models that can support biomedical innovation. The research questions also explore the impact of crowdfunding on portfolio risk, innovation translation, entrepreneurship skills, and healthcare systems.

### Cluster 2: Innovation in medical technology (n = 23; 29.11%)

The second cluster examines the challenges and opportunities of innovation in the medical technology field. The articles explore various aspects of the innovation process, such as the usage and benefits of mHealth, the impact of demand-side shocks, the role of regulatory knowledge, the process of life science investment, and the framework of open innovation.

### Cluster 3: Study on new product development (n = 17; 21.52%)

The third cluster analyses factors and strategies that influence the success of new product development in the pharmaceutical and medical device sectors. A range of associated topics are covered in example: networking activities, telehealth business frameworks, industry-academia collaboration in medical device development, commercialization, convergence of pharmaceutical and medical device sectors, techno-entrepreneurship and decision-making processes.

### Cluster 4: Digital start-ups (n = 10; 12,66%)

The fourth cluster explores the potential and challenges of digital start-ups in healthcare. Included articles are devoted to understand the context of ventures emerging and operating in the healthcare and telemedicine sectors, including their development trajectory, connected health concepts, clinical robustness and claims, managerial experiences, quality management systems for medical device start-ups, and value proposition and support for biomedical start-ups.

### Cluster 5: Venture capital industry (n = 14; 17.72%)

The last cluster cover a wide range of topics in the healthcare industry that is in the scope of interest of Venture Capital. This included venture capital investments, time to IPO for VC-backed companies, capital funding and adoption challenges for university spin-outs in biomaterials and medical devices, the probability of venture loans for start-ups in various sectors, and the influence of institutional logics on firms’ innovation search.

### Cluster analysis

The following section provides a detailed description of the content of each of the clusters. This analysis aims to elucidate the key themes, findings, and implications of the research within each cluster, offering a comprehensive understanding of the current state and future directions in various areas of medical research and innovation. By examining these clusters, we can identify critical insights and trends that are shaping the landscape of medical technology, healthcare innovation, and funding mechanisms.

### Cluster 1—Crowdfunding for medical research

Cluster 1 (Crowdfunding for medical research) is relatively homogeneous in respect to the discussed issues. The predominant focus is given to crowdfunding as a novel tool allowing collection of funds that are difficult to obtain though other means.

Authors indicate the unique and growing role of crowdfunding in the global health sector. Benazzouz et. al. [19] study on neglected tropical diseases reveals that although the number of crowdfunding campaigns in this area has been increasing since 2010, crowdfunding has not yet reached its full potential. They indicate the need to promote the crowdfunding ecosystem for research and development of treatments for these diseases. This statement is supported by Tóth et al. [20] research focusing on the potential of crowdfunding for funding research on incurable diseases such as Alzheimer’s and Parkinson’s. Crowdfunding is perceived as a promising mean to increase funding for the development of diagnostics and therapies and overall advancing medical research and development. Dal-Ré et al. [21] suggest future scenarios where crowdfunding could enable self-funded clinical trials in Spain. Yet, earlier efforts into education of both patients and researchers on the crowdfunding mechanisms is crucial in order to unlock its full potential.

Further, articles explored factors responsible for the success of crowdfunding campaigns, such as social media coverage, public engagement, testimonials and inputs of known NGO’s or disease characteristics [18, 22]. Both potential benefits and risks of crowdfunding for health research are discussed. Expanding market participation, improving access to funding, fostering project accountability are elicited on the benefit side, yet inefficient priority setting, heightened financial risk, inconsistent regulatory policies, as well as fraud balance them on the risks side [23]. The discussed papers also investigate crowdfunding initiatives from the perspective of different types of medical research, for example therapies for rare diseases [21], diagnostics for neglected tropical diseases [19] non-invasive methods to visualize neuronal fibre tracts or securitization techniques for biomedical innovation [24].

A fraction of the studies included in the cluster indicates innovation culture, training programs, and collaborations as a need for advancing healthcare research and development. Young’s [25] findings emphasize the importance of developing an innovation culture within the healthcare system. This involves empowering multidisciplinary teams and reducing the time from invention to adoption of innovative solutions. By fostering an environment that embraces innovation, the healthcare sector can realize substantial benefits and drive economic growth. Whereas, Nearing et al. [26] research highlights the value of training programs like I-Corps™ in supporting biomedical researchers. These programs focus on developing business models based on customers’ needs, with the ultimate goal of accelerating research translation into practice. Finally, Germann et al. [27] explore different aspects of collaboration between universities and the pharmaceutical industry. Concluding that trust-building and effective translation of innovations into the value chain of pharmaceutical companies are the crucial phases for an effective collaborative process.

Fernandez et al. [28] examine a broader context of funding models for biomedical research. The authors propose financial engineering as an alternative approach, which involves employing techniques like securitization to raise larger amounts of capital. They also suggest that creating diversified portfolios and combining equity and debt financing, will lead to attract significant investments from institutional players.

In conclusion papers classified in cluster one suggest that crowdfunding can be a promising and feasible alternative source of capital for medical research, yet it also requires careful design and implementation to optimize and equitably improve public health outcomes [19, 21, 23, 24]. In conclusion, papers in cluster one suggest that crowdfunding is a promising alternative source of capital for medical research, requiring careful design and implementation to optimize and equitably improve public health outcomes [23].

### Cluster 2—Innovation in medical technology

Cluster 2 (Innovation in medical technology) focus on several interconnected themes, such as innovation, new technologies and commercialisation challenges. The research findings emphasized the importance of collaboration, innovation, effective management, and strategic considerations for the success of medical start-ups and the advancement of the medical device industry.

The literature agreed that commercialisation of medical technology requires a long development period and bears a high risk of failure for the ventures [29]. Thus a range of suggestions how to overcome those key challenges is proposed.

Kalcheva et al. [30] highlight the importance of collaboration between universities, industry, and government, arguing that the blend of different knowledge and expertise increases the chances of commercialization success Lottes et al. [31] add to the discussion by emphasising the need for collaboration and cooperation among clinicians, researchers, industry, and regulators for successful translation of innovation into medical products, thus indicating the communication between stakeholders as the critical factor. Wagrell and Baraldi [32] and Onodera and Sengoku [33] provided illustration to the above statements by pointing out the significant influence of new entries and start-ups in shaping the mHealth industry through expertise in information and communication technology.

Considering identified factors contributing to success and failure of commercialisation, Wagrell and Baraldi [32] pointed out the regulatory barriers, complex decision-making processes, and customer-related challenges for start-ups. Effective management of knowledge flow, integration of regulatory requirements and addressing unmet needs are elicit as key success factors of medical device start-ups by Wan and Quan [34]. The factor list is enriched by Ren and Wu [35] who indicated the importance of environmental friendliness and effective communication in fundraising success in the medical products industry. Challenges in forming collaboration networks were in scope of interests of Moazzez et al. [36], who also provided models and strategies for network formation.

Finance and investment strategies are also discussed. Keppler et al. [37] described new investment criteria developed by the Venture Capitals for assessing the medical technology sector. The entrepreneurial environment created for medical device and biotech companies through leveraging resources was discussed by Hafer et al. [29]. Whereas, Alexander et al. [38] identified technology clusters with growth prospects in medical imaging and explores their implications.

Another sub-theme, that could be identified refers to the role of Open Innovation (OI) within the medical technology industry. OI had been indicated as an efficient strategy to obtain outside knowledge and thus enhance ones capabilities, or share unused knowledge either for free or fee. The identified papers focus on OI practices, OI drivers and OI barriers utilizing case studies as illustrations [34, 39].

### Cluster 3—Study on new product development

Papers in this cluster focus on product development in Med-Tech, specifically drivers for innovation, medical device development and commercialization, and capital investment.

In the realm of Drivers for innovation Cummings et. al. [40] highlight the importance of financial support and strategic partnerships. Whereas Laurell [41] brought attention to the significance of local network ties in the development of innovative products and the establishment of companies during early development phases. The study underscored the role of key individuals within the local network in fostering innovation and driving the growth of med-tech ventures. Also Kikuchi (2021) reveals that companies with high network centrality in alliances are more competitive and likely to develop products, underscoring the importance of strategic alliances. Building on this, Amano-Ito [42] shed light on the importance of industry-academia collaborations in the medical device field. The study emphasized the need for interventions by collaboration coordinators to ensure successful collaborations, as failures often arise from a lack of understanding of marketing and technical needs. Moreover, Hasche and Linton [43] pointed out that forming relationships and a strong network may help start-ups to overcome the liability of newness and smallness common for early stage of development.

Several observations had been made in reference to the Medical Device Development and Commercialization aspects. Bhaskaran [44] explores optimal product introduction strategies, arguing that start-ups may benefit from delaying launches while established firms should accelerate high-quality product launches. Velayati [45] emphasized the critical need for formulating a telehealth business framework that facilitates commercialization and ensures long-term sustainability in a competitive market. Identifying key components and critical factors for developing such a framework becomes essential for med-tech companies to thrive. Rose [46] highlighted the positive impact of programs like the Drug and Device Advisory Committee (DDAC) on the commercialization metrics of medical product investigators. The formalized partnerships between preclinical consulting and technology transfer programs enhance development and regulatory vetting, driving the translation of discoveries into tangible medical products.

Considering the discussion on Capital Investment and Innovation Influence, issues reflecting the strategies and methods applied by the stakeholder to assess the risks and business opportunities related to the new ventures are discussed. Lehoux et. al [47] examine capital investors’ decision-making processes in health technology ventures, focusing on how they assess, build, protect, and monetize value, thereby shaping innovation. On the other hand DiLorenzo [48] proposes a new metric–social return on investment–that may be utilised while assessing new ventures.

In conclusion, studies collected within this cluster contribute to a better understanding of the wide range of processes undergoing in the area of innovation and commercialization of medical devices within the industry.

### Cluster 4—Digital start-ups

Papers in this cluster, titled "Digital Start-ups," focus on digital health innovation and start-ups. We identified three thematic groups: technological convergence, clinical robustness and public claims, and experiences of start-up managers.

The first theme covers research discussing the notion of technological convergence. Both, Livi and Jeannerat [49] and Chen et al. [50] provided evidence and indicate the tendency of technological convergence in the industry. They emphasize the need for continuous innovation and adaptation to stay ahead in the medical field, which can be achieved by combination a strong demand for innovation paired with continuous patenting. Observations made by Saarela et al. [15] indicated that market behaviour as well as the innovation strategies undertaken by companies differ depending on their size. The small companies are perceived to create more new to market products, yet the middle and large size companies specialise in spreading the innovations already known to market.

Although, the digital health sector experiences a significant growth, the clinical validation of products remains underdeveloped, which is discussed within the second leading theme. The evidence provided by Day et al. [51] indicated that many of digital health companies have a low level of clinical robustness and do not make many clinical claims measured by regulatory filings, clinical trials or public data shared on line. Yet, authors suggested that investing in greater clinical validation efforts can benefit both companies and customers, ensuring the credibility and reliability of digital health solutions. Ferguson [52] pointed out in order to successfully commercialise novel basic and clinical research into market products laboratories and universities need to seek a corporate partner or licensees.

The third theme focuses on the experiences of start-up managers. Saarela et al. [13] use a stage framework to identify the challenges faced by start-up managers, providing insights into the start-up growth stage in digital health. The traditional model of territorial innovation is challenged, with Swiss experience showing that med-tech companies are often shaped by multinational corporate venture strategies, classifying many as ’born to be sold’ [49] In contrary, the lack of quality management strategies and issues related to challenges of growth management are key factors hindering development of digital health start -ups [53].

In conclusion, research classified under this cluster shed light on the current state of digital health innovation and emphasizes the importance of continuous innovation, clinical validation, and understanding the unique challenges faced by start-ups in this dynamic industry. Despite the wide range of issues discussed the literature indicates that terms such as medical informatics, e-health, telemedicine or m-health, which are traditionally associated with digital health remain blurred and require further systematisation [50]. Moreover, the majority of the presented results derive from qualitative research and is based on either single, or multiple case studies.

### Cluster 5—Venture capital industry

Cluster 5 contains research focusing both on venture capital and corporate venture capital in the context of med-tech industry. Studies in this cluster highlight various aspects of the healthcare innovation ecosystem, including venture financing, institutional factors, technological innovations, industry-specific challenges, and ecosystem development.

In terms of venture financing, researchers have explored various factors influencing the financing decisions of healthcare startups. Khanin et al. [54] study reveals that higher patent counts, as an indicator of national system of innovation advancement, are negatively associated with the likelihood of initial public offerings (IPOs) for VC-backed foreign portfolio companies. This finding suggests that a greater emphasis on patent generation may not necessarily translate into successful IPOs in the healthcare industry. Another aspect of venture financing is the use of venture loans by startups. Lehnertz’ et. al. [55] research showed that venture loans are more likely for mature start-ups with strongly committed existing investors. These loans are particularly common in the medical, health, and life science industry, where clear milestones provide favourable conditions for venture capital investments. This finding highlights the importance of investor commitment and the stage of start-up development in securing venture loans. While analysing Institutional Factors and Partnerships, Pahnke et al. [16] research explored how the institutional logics of funding partners influence the innovation efforts of young firms. The study revealed that ties with specific types of funding partners lead to significant differences in innovation outcomes, even when partners provide relevant resources, selectivity, and incentives for innovation. This highlighted the importance of considering the alignment of institutional logics when forming partnerships in the healthcare industry. Those findings are supported by Pei and Dang [56] study that examined the impact of venture capital network community structure on information dissemination in the medical health industry. The research showed that different types of network community structures can either promote or hinder the flow of information, ultimately affecting the sustainable development of the industry. Understanding these dynamics can help stakeholders shape network structures that foster effective knowledge exchange and collaboration. Whereas, Shakeri and Radfar [57] research focused on the performance of strategic alliances for technology commercialization in the biopharmaceutical industry. The study highlighted the determinants of alliance performance, including partner fit, alliance capabilities, social capital, and learning. It emphasized that opportunistic behaviour negatively affects alliance performance, while factors such as partner fit, conflict management, trust, and social capital positively influence learning and, consequently, alliance outcomes.

Technological innovations are also crucial. Smith and Shah [58] research demonstrated that established firms derive greater innovation-related benefits by incorporating user knowledge in the development of new technologies and products. This highlights the importance of user-driven innovation approaches in the healthcare industry. Levin and Behar-Cohen [59] study reveals disruptions in the flow of information in the pharmaceutical industry due to different goals among stakeholders. This finding suggests the need for improved communication and collaboration among stakeholders to enhance the reproducibility of academic laboratory results and promote innovation in the pharmaceutical sector.

The impact of Industry 4.0 technologies, such as the Internet of Things (IoT), Big Data Analytics (BDA), blockchain, Artificial Intelligence (AI), and cloud computing, on the healthcare industry was discussed by Paul et. al. [60]. The study highlighted the transformative potential of these technologies and their applications, challenges, and overall impact on shaping the future of healthcare.

Specific industry challenges include the adoption issues faced by university spin-outs in biomaterials and medical devices [61]. The research suggests that there may be an overemphasis on early-stage support, while more mature companies in this sector lack unified regulatory frameworks and policies for the procurement of healthcare innovations. Addressing these challenges can foster the growth and success of biomaterials and medical device companies [61]. Lehoux et al. [62] work explores the evolution methods of health technology spin-offs, providing insights into this specific industry context. Understanding the trajectory of health technology spin-offs can help stakeholders better navigate the landscape and develop effective strategies for innovation and commercialization.

The last theme—ecosystem development and funding—encompasses studies that examine the broader environment and support mechanisms for healthcare innovation. DiLorenzo [48] emphasized the importance of factoring in the societal return on investment (sROI) in decision-making for neurotechnology and therapeutic technologies. By considering the broader societal impact, policymakers and investors can prioritize technologies that deliver the greatest benefit to patients and society. Williams and Walch [63] study highlighted the lack of available financial capital, particularly venture capital, as a major hindrance to ecosystem development, especially in Austrian regions. It underscores the critical role of venture capital in fostering biopharmaceutical innovation ecosystems. Increased government funding, including public grants, is essential for areas lacking venture capital to foster innovation and support the growth of biopharmaceutical companies.

In conclusion, the studies presented in these thematic categories contribute to the insights into the field of healthcare innovation. They offer a comprehensive understanding of venture financing and investment, the role of institutional factors and partnerships, the impact of technological innovations, the challenges and opportunities in specific industry sectors, and the importance of ecosystem development and funding. By addressing various industry specific aspects, stakeholders can make informed decisions, foster collaboration, and drive innovation to enhance healthcare delivery and outcomes.

### Research gaps based on the clusters analyses

A scoping literature review was conducted to identify underexplored areas within the MedTech field and to formulate critical questions for future research. Table 4 presents research questions inspired by previous studies, each highlighting specific research gaps that need to be addressed for a better understanding of the MedTech industry. These questions aim to guide future research efforts, ensuring they build upon the existing knowledge base and address unresolved critical issues.

**Table 4:** Future studies in the clusters.

Clusters	Future studies
*1*: *Crowdfunding for medical research*	**Crowdfunding for Research and Development (R&D) in medical Innovation:** How can the expanding phenomenon of crowdfunding for health research be assessed, and what are the implications for funding and research outcomes? [22] What is the significance of crowdfunding in financing research related to tropical diseases, and how does it compare to other funding sources? [19] **Determinants and Outcomes of Crowdfunding in Medical Research** What is the post-crowdfunding performance of scientific and technological ventures, and how does crowdfunding impact science in general? [24]
*2*: *Innovation in medical technology*	**Importance of Collaboration and Innovation:** What are the challenges in finding, forming, and performing in collaboration networks? Can we rank and prioritize these challenges quantitatively? How do these challenges interdependently affect each other? [36] **Innovation in medical start-ups:** Which technologies will emerge as winners in the medical technology field? How can we determine their value and adoption rates? What clinical outcomes evidence is available, and what additional evidence do we need? [38] Is the speed of technological innovation declining in recent times? Can we model more accurate and reliable periodic patterns of technological innovation? [64] **Factors Influencing Success in Medical Start-ups:** Do interactions with the public sector as a customer in the medical technology industry predominantly result in challenges rather than benefits? How does the role of public actors impact a start-up’s growth? Is this pattern observed in other industries as well? [32] **Open innovation for medical technology:** How do various actors interact in the open innovation process of medical technology? What is the complex interplay among these actors? [34]
*3*: *Study on new product development*	**Optimal strategy for developing innovation in med-tech start-ups** What are the optimal product introduction strategies for startups and established firms? [44] How does the impact of technological failure and different forms of learning influence the optimal strategy for startups? [44] How can startups in the pharmaceutical and medical devices industries effectively develop and launch new products? [44]
*4*: *Digital start-ups*	**Leveraging ICT Solutions and Innovating Operation Models in Healthcare** What are the potential impacts and benefits of implementing telehealth services and ambient assisted living research programs in healthcare? [50] How can new ICT solutions be effectively utilized in various aspects of acute and chronic clinical care? [50] **Cross-Country Start-up Growth Management and Network Management Challenges** How do growth management priorities differ in the start-up process across different countries, and what factors contribute to these variations? [14] What specific forms of support do business ecosystems provide to start-ups, and how do they facilitate their growth and success? [14] What are the key considerations and challenges in network management as a context-specific management priority for digital service start-ups, and how can these barriers be addressed effectively? [14]
*5*: *Venture capital industry*	How significant are patents for venture lenders, and what is the impact of heterogeneity among different venture debt vehicles? [55] How do institutional factors affect venture IPOs, and what are the relationships with venture-specific resource endowments and VC financing and support methods? [54] How can cost-effective innovation be ensured while balancing orthopedic-industry growth and prioritizing patient safety? [65]

To summarize, in Cluster 1 (Crowdfunding for Medical Research) the future research directions should focus on refining mapping and quantification methods, evaluating the long-term impact of crowdfunding on research outcomes, and examining the broader implications of crowdfunding for the scientific community.

Cluster 2 (Innovation in Medical Technology) suggests to further explore methods for evaluating the value and adoption rates of emerging technologies, identify strategies to overcome challenges in collaboration networks, and assess the role of public actors in fostering innovation across different industries.

Cluster 3 (Study on New Product Development) indicates the need to develop effective product launch strategies, understanding the dynamics of drug-device convergence, and exploring decision-making processes in innovation-driven ventures.

Cluster 4 (Digital Start-ups) advocates for evaluating the outcomes and benefits of telehealth and ICT solutions, understanding the dynamics of co-creation practices in different sectors, and identifying effective strategies for managing growth and networks in digital start-ups.

Finally, Cluster 5 (Venture Capital Industry) recommends to focus on assessing the impact of patents on venture lending decisions, developing strategies for cost-effective innovation while ensuring patient safety, and exploring alternative outcome variables and knowledge sources to provide a comprehensive understanding of the benefits in the venture capital industry.

## Conclusion

The scoping review on MedTech start-ups presents a multifaceted exploration of the current research landscape, highlighting significant insights and identifying critical gaps in the literature. Despite the consensus among scholars on the importance of this research area, the analysis described in the article reveals a limited number of publications. However, there is a noticeable increase in interest and a growing trend in the number of studies being published on the topic.

The review identified several major limitations in the existing research. One of the most striking findings is the geographic concentration of research, with a significant portion of studies centered in specific countries, notably the United States. This concentration highlights a gap in the literature regarding the experiences and developments in other regions, suggesting a need for more inclusive research that encompasses a broader range of healthcare contexts and markets globally. Expanding the geographic scope of research could provide a more comprehensive understanding of the global MedTech landscape and uncover region-specific insights that could inform more tailored and effective innovation strategies.

Methodologically, the review found that most studies employ qualitative approaches, such as case studies and in-depth interviews. Consequently, the research samples were typically small, limiting the scope and generalizability of the findings. Only a few studies involved larger samples (above 100) and utilized advanced econometric models. While qualitative methods provide rich, contextual insights into the MedTech ecosystem, the reliance on small sample sizes and qualitative data points to a need for more large-scale quantitative research. Despite the challenging nature of studying start-ups, particularly within a specific sector such as MedTech, there is potential for enriching the current qualitative research with more sophisticated quantitative research methods based on larger samples in future studies. Such research could enhance the generalizability of findings and offer more robust statistical analyses, thereby providing a clearer picture of trends and patterns within the industry.

The research revealed that the MedTech ecosystem is characterized by five predominant thematic clusters: crowdfunding for medical research, innovation in medical technology, new product development, digital start-ups, and the venture capital industry. Each cluster offers a unique perspective on the challenges and opportunities within the MedTech field, underscoring the diversity of research interests and the complexity of the ecosystem.

The commercialization of medical technologies emerged as a significant challenge, with regulatory barriers, complex decision-making processes, and customer-related issues frequently cited as obstacles. Effective collaboration among stakeholders—including universities, industry players, and government bodies—is essential for overcoming these hurdles. By fostering robust collaboration networks and integrating regulatory requirements early in the development process, MedTech start-ups and established firms alike can enhance their chances of successful innovation and commercialization.

The role of crowdfunding and venture capital in supporting medical research and innovation was another critical area of focus. Crowdfunding is increasingly seen as a promising alternative funding source, particularly for early-stage projects that may struggle to attract traditional investment. However, the high risks associated with medical innovation and the stringent regulatory environment pose significant challenges for start-ups seeking to bring new technologies to market. Venture capital remains a crucial driver of innovation, with significant interest in cutting-edge technologies such as artificial intelligence and robotics. To mitigate risks and enhance the success rate of funded projects, investors should consider strategies that include diverse portfolios and strong support for regulatory compliance.

The implications of these findings for various stakeholders are profound. For industry practitioners, it is essential to emphasize robust collaboration among different stakeholders, including researchers, healthcare providers, and regulatory bodies. Early integration of regulatory requirements into the development process and a keen focus on addressing customer needs are crucial for the successful commercialization of medical products. Investors, including venture capitalists and crowdfunding platforms, should develop strategies that mitigate the high risks associated with medical innovation. This involves supporting projects that have clear regulatory pathways and demonstrating strong potential for market success, ensuring that investments are both secure and impactful in advancing healthcare technology.

Future research should aim to address the identified gaps through more inclusive, quantitative, and longitudinal studies. Expanding research to underrepresented regions, employing advanced statistical techniques, and tracking long-term outcomes are essential steps towards a more comprehensive understanding of the MedTech ecosystem. Interdisciplinary approaches that incorporate perspectives from economics, sociology, and business could also enrich the field, providing a more holistic view of the challenges and opportunities in MedTech innovation. Additionally, the research questions presented in the article for each cluster can be utilized in future studies.

The presented review has its limitations. Although data was integrated from three distinct databases, certain bibliometric information, such as details on research funding sources, remained inaccessible. Additionally, the study did not include grey literature, such as reports and industry publications, which could offer valuable insights into the latest developments within the medical start-up landscape.

We assume there might be a delay between new industry trends and the corresponding academic research. This lag can be attributed to the research cycle, which requires time for identifying new phenomena, collecting data, analyzing it, and publishing the results. Consequently, our article can serve as a foundation for future studies that compare academic literature with grey literature. While we identified research topics in academic studies, it would be interesting to compare them with the themes found in grey literature. This comparison could enhance our understanding of the MedTech industry and suggest directions for future research.

In conclusion, the scoping review offers a detailed map of the current research landscape on MedTech start-ups, highlighting significant themes, challenges, and gaps. Addressing these gaps through more inclusive, quantitative, and interdisciplinary research can significantly advance the field, informing policy, practice, and investment strategies. By fostering collaboration and leveraging emerging technologies, the MedTech industry has the potential to drive innovations that enhance healthcare outcomes globally.

## Supporting information

S1 Data (XLSX)

S1 File (DOCX)
