# Supplementary material for: MedTech start-ups: A comprehensive scoping review of current research trends and future directions
Source: PLoS One. 2024 Aug 6;19(8):e0307959. doi: 10.1371/journal.pone.0307959 (PMC11302850; doi:10.1371/journal.pone.0307959)
Supplement: S1 File — (DOCX) [file pone.0307959.s002.docx]

MedTech start-ups: a comprehensive scoping review of current research trends and future directions

Olga Kalinowska-Beszczyńska^1*^, Katarzyna Prędkiewicz^2^

# ^1^ Division of Public Health & Social Medicine, Medical University of Gdansk, Gdansk, Poland

^2^ Finance Department, Wroclaw University of Economics and Business, Wroclaw, Poland

*Corresponding author

Email: [o.kalinowska-beszczynska@gumed.edu.pl](mailto:o.kalinowska-beszczynska@gumed.edu.pl) (OKB)
